# Supplementary material for: Comparison of Venous and Capillary Sampling in Oral Glucose Testing for the Diagnosis of Gestational Diabetes Mellitus: A Diagnostic Accuracy Cross-Sectional Study Using Accu-Chek Inform II
Source: Diagnostics (Basel). 2020 Nov 26;10(12):1011. doi: 10.3390/diagnostics10121011 (PMC7760160; doi:10.3390/diagnostics10121011)
Supplement: Supplementary file 1 [file diagnostics-10-01011-s001.pdf]

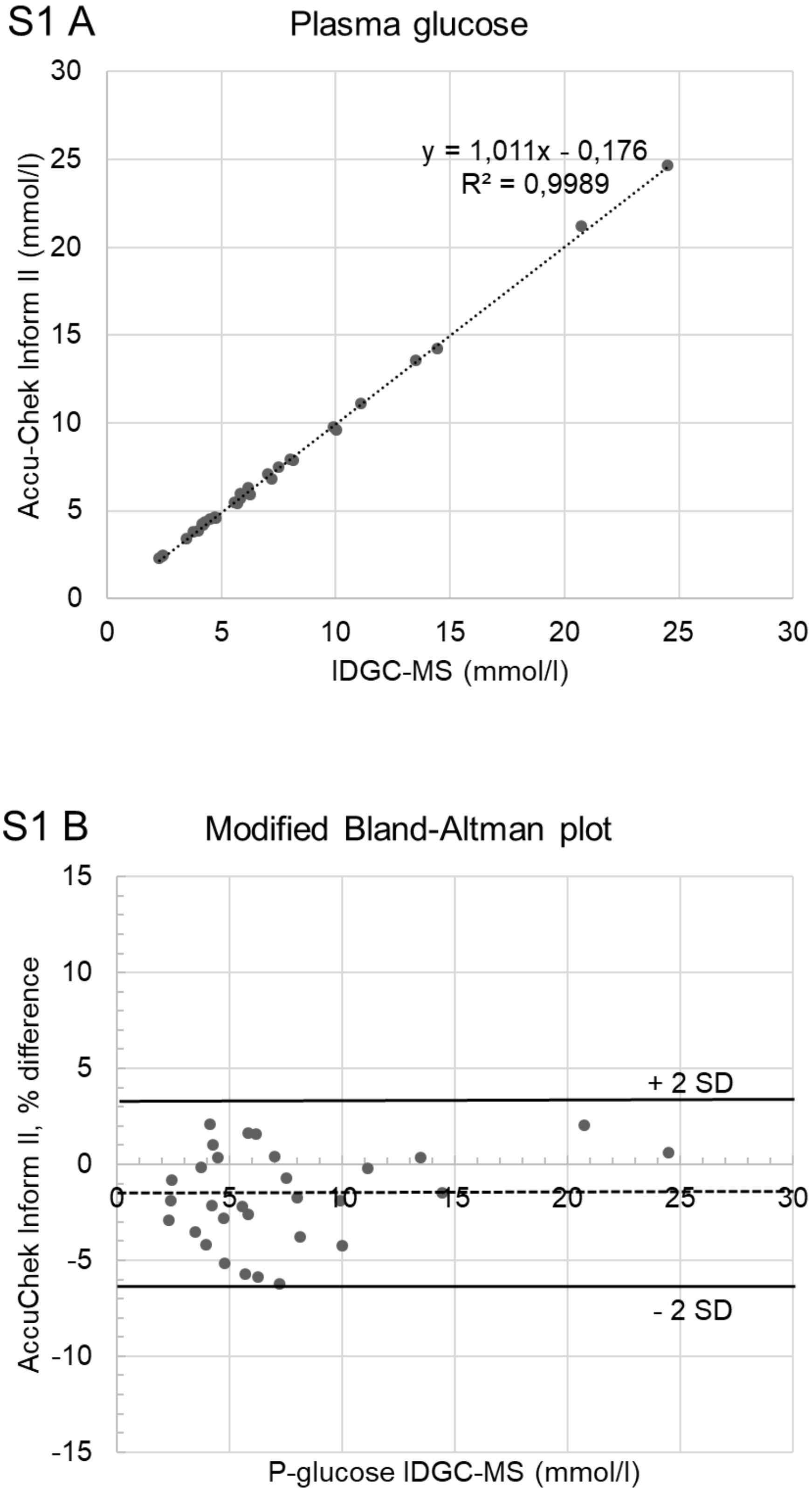

Figure S1. Validation of Accu-Chek Inform II against IDGC-MS reference method. Results represent mean values from four instruments and duplicate analyses on each instrument.
